# International aid dependence and healthcare sustainability in Afghanistan: study protocol for a systematic review

**DOI:** 10.1136/bmjopen-2026-117725

**Published:** 2026-11-04

**Authors:** Ramim Rahman, Seyed Aria Nejadghaderi, Mohammad Sharif Razai

**Affiliations:** 1 Department of Public Health and Primary Care, University of Cambridge, Cambridge, UK; 2 HIV/STI Surveillance Research Center, and WHO Collaborating Center for HIV Surveillance, Institute for Futures Studies in Health, Kerman University of Medical Sciences, Kerman, Iran; 3 Knowledge Hub for Migrant and Refugee Health, Institute for Futures Studies in Health, Kerman University of Medical Sciences, Kerman, Iran

**Keywords:** PUBLIC HEALTH, HEALTH ECONOMICS, HEALTH SERVICES ADMINISTRATION & MANAGEMENT, Primary Health Care

## Abstract

**Abstract:**

**Introduction:**

Afghanistan’s healthcare system has been heavily dependent on international aid for financing, service delivery and workforce support. Following substantial reductions in international support, particularly since 2021, many health facilities have closed or experienced severe shortages, with reported declines in access to care and worsening population health outcomes. While the deterioration of the system has been described, it remains unclear which components of the health system have been most affected and how these changes have translated into health outcomes across population groups. This systematic review aims to synthesise evidence on the effects of reduced international aid on healthcare system functioning and population health outcomes in Afghanistan.

**Methods and analysis:**

The systematic review protocol was developed and reported in accordance with Preferred Reporting Items for Systematic review and Meta-Analysis Protocols (PRISMA-P) guidance. We conducted a comprehensive search of MEDLINE, Embase, Global Health and Web of Science from January 2000 to January 2026 and searched relevant organisational websites and Google Scholar for grey literature from January 2000 to June 2026. Eligible studies will include qualitative, quantitative and mixed-methods primary research focused on health systems or population health in Afghanistan. Study selection, data extraction and critical appraisal will be undertaken independently by two reviewers. Study quality will be assessed using the Mixed Methods Appraisal Tool (MMAT), and certainty of evidence using the Grading of Recommendations, Assessment, Development and Evaluation (GRADE) framework. Findings will be synthesised using a narrative synthesis, structured around the WHO health system building blocks, with subgroup analyses to explore variation across population demographics where data permit.

**Ethics and dissemination:**

This study does not require ethical approval as it involves secondary data analysis. Findings will be disseminated through publication in peer-reviewed journals.

**PROSPERO registration number:**

CRD420261371072.

**STRENGTHS AND LIMITATIONS OF THIS STUDY**

- This review uses a system-wide framework to examine how reductions in international aid affect specific health system components and associated population health outcomes in Afghanistan.
- Inclusion of qualitative, quantitative and grey literature allows the review to capture outcome data and operational evidence from a fragile and conflict-affected setting.
- Heterogeneity and uneven evidence across health system domains and population groups, particularly after August 2021, may limit synthesis for some outcomes.
- Findings may have limited direct applicability beyond Afghanistan, although system-level patterns identified may be relevant to other aid-dependent health systems.

## Introduction

 Dependency on international aid is defined as a situation in which at least 10% of a country’s Gross Domestic Product (GDP) is derived from international support, and without this aid, the government would be unable to perform core functions.^1^ In 2021, international aid accounted for 43% of Afghanistan’s GDP.^2^ Additionally, 19.4% of total healthcare expenditure in Afghanistan was funded by external partners and donors^3^ such as the European Union (EU) and the International Monetary Fund (IMF),^2^ significantly higher than neighbouring Pakistan’s 0.4%.^4^ Beyond financial support, international aid also included technical and humanitarian assistance,^5^ such as medical resources.^6^ This over-reliance on international support for healthcare was evident not only at the system level but also at the population level. The externally funded Basic Package of Health Services (BPHS) and Essential Package of Hospital Services (EPHS) provided comprehensive services for maternal care, childcare and immunisation.^7^ Through these programmes, healthcare coverage of the population increased from 12% in 2001 to 90% in 2021.^2^ As a result, mortality among children under 5 years of age decreased by 29% between 2003 and 2015.^8^ Similarly, childhood vaccination coverage increased rapidly between 1999 and 2006, before increasing gradually from 2007 to 2018.^9^ These increases coincided with large influxes of international funding.

The consequence of international aid dependency is an unsustainable healthcare system.^10^ Sustainability is defined as when, without external support, a healthcare system displays three properties: (1) the ability to enhance population health throughout changing disease landscapes; (2) adaptability to new leadership or organisational structures and (3) equitable access and service of the whole community.^11^ After the Taliban came to power in August 2021, international donors withdrew or suspended funding,^2^ exposing the system’s structural inability to function independently of external support.

By September 2021, fewer than 20% of healthcare facilities were operational.^12^ Facilities still in operation faced financial and equipment constraints that restricted their ability to provide care.^6^ Access to healthcare has also drastically declined, as 43% of the Afghan population do not have access to primary healthcare.^13^ Women, particularly in rural areas, have experienced major challenges to accessing care, as limited availability of BPHS services, alongside restrictive laws on female employment, has severely reduced maternal care provisions.^14^

This system-wide contraction, coupled with increasing levels of poverty,^15^ has had severe effects on population health outcomes. In 2022, an estimated 18.1 million people needed urgent health services,^16^ while the number of people in need of humanitarian aid reached nearly 25 million—more than half of Afghanistan’s estimated population of 45.2 million.^17 18^ Core vaccination coverage for children also declined between 2018 and 2023.^9^

The recent breakdown of Afghanistan’s healthcare system demonstrates the risks of building a fragile healthcare system, overly reliant on external support.^19^ Emergency funding from the United Nations (UN) and the Global Fund is providing temporary aid,^20^ supporting those most in need. Thus, identifying which health system domains have been most affected by reduced aid and the associated population health effects is important for informing future resource allocation.

Much of the academic literature has focused on documenting the deterioration of Afghanistan’s healthcare system, with reductions in international support identified as a key driver.^21^ However, it remains unclear which components of the health system have experienced the greatest adverse effects and how these disruptions have translated into changes in health outcomes across population groups.

### Objectives

This systematic review aims to fill this gap by synthesising evidence from a range of sources on the effects of reductions in international aid on health system functioning and population health outcomes across population groups.

## Methods

### Reporting guidelines

This study will be conducted as a systematic review. The protocol is registered with the International Prospective Register of Systematic Reviews to maintain scientific integrity. The protocol is in accordance with the Preferred Reporting Items for Systematic review and Meta-Analysis Protocols (PRISMA-P) guidelines.^22^

### Research questions

This study aims to synthesise evidence on how reductions in international aid have affected the capacity of Afghanistan’s healthcare system to deliver care and associated health outcomes. The specific research questions are as follows: (1) How did international aid impact Afghanistan’s healthcare system and population health outcomes between 2000 and 2021? (2) How did the Taliban’s return to power in August 2021 affect international aid provision, and how did this affect the health system and population health outcomes? (3) What strategies have been reported to mitigate the effects of reduced international support and strengthen the sustainability of Afghanistan’s healthcare system?

### Eligibility criteria

Given the exploratory, mixed-methods nature of this review, the Sample, Phenomenon of Interest, Design, Evaluation and Research type (SPIDER) framework was used to define search terms and eligibility criteria.^23^ Table 1 presents the SPIDER framework applied to the research questions.

**Table 1:** SPIDER framework applied to research questions

SPIDER component	Description
Sample	Afghanistan’s healthcare system and population groups, including health facilities, health workers and service users across demographic groups
Phenomenon of Interest	Reductions in international aid (financial and non-financial), including funding, staffing, supplies, infrastructure and technical support
Design	Qualitative, quantitative and mixed-methods primary studies, including observational studies, surveys, routine data analyses and qualitative interviews
Evaluation	Effects on healthcare system functioning (mapped to WHO health system building blocks) and population health outcomes (eg, mortality, morbidity and service coverage) Strategies reported to mitigate the effects of reduced international aid
Research type	Qualitative, quantitative and mixed-methods studies

SPIDER, Sample, Phenomenon of Interest, Design, Evaluation and Research.

### Information sources

We conducted a search of MEDLINE via Ovid, Embase via Ovid, Global Health and Web of Science from January 2000 to January 2026. Additionally, we searched websites of the Afghanistan Ministry of Public Health, Afghanistan Research and Evaluation Unit, WHO Global Index Medicus, World Bank and UNICEF, as well as Google Scholar from January 2000 to June 2026 to identify relevant grey literature. The searches were undertaken sequentially. Screening of the database records informed the subsequent searches of organisational websites and Google Scholar by clarifying the types of reports most relevant to the review. Conducting the grey literature search later also allowed us to identify more recent relevant organisational reports.

### Search strategy

The search strategy was developed with the support of a medical librarian using the SPIDER framework.^23^ Full search strategies for each database are provided in online supplemental file 1.

After deduplication, 1271 records remained for screening. Records were managed using Covidence^24^ and all references were stored in EndNote.

### Study selection

The inclusion criteria and their justification are presented in table 2. Conference abstracts, commentaries, opinion articles, editorials and review articles will be excluded.

**Table 2:** Inclusion criteria and justification

Category	Inclusion criteria	Justification
Source	Peer-reviewed articles or grey literature reports	To capture both academic and operational evidence relevant to health system functioning in a humanitarian and conflict-affected setting
Study design	Any primary qualitative, quantitative and mixed-methods studies	To allow synthesis of diverse forms of evidence suitable for investigating population- and system-level effects of aid reductions
Focus	Focuses on health and the healthcare system	To ensure included studies directly relate to healthcare provision rather than broader political or economic analyses
Population and setting	Afghanistan’s healthcare system, including health facilities, health workers and population groups	Afghanistan represents both the geographic focus and the population of interest due to its high reliance on international aid
Analytical relevance	Findings reported by healthcare system components and/or population demographics	To enable analysis of differential effects across system domains and demographic groups
Timeframe	Published from January 2000 onwards but especially after August 2021	To capture long-term aid dependence trends and the effects of the most recent disruptions^37^
Language	Full text available in English	To ensure consistent independent screening, data extraction and appraisal across reviewers

Each article will be screened independently by two reviewers (RR and MSR) using a two-stage process, as outlined by the PRISMA flow diagram (figure 1).^25^

**Figure 1:**
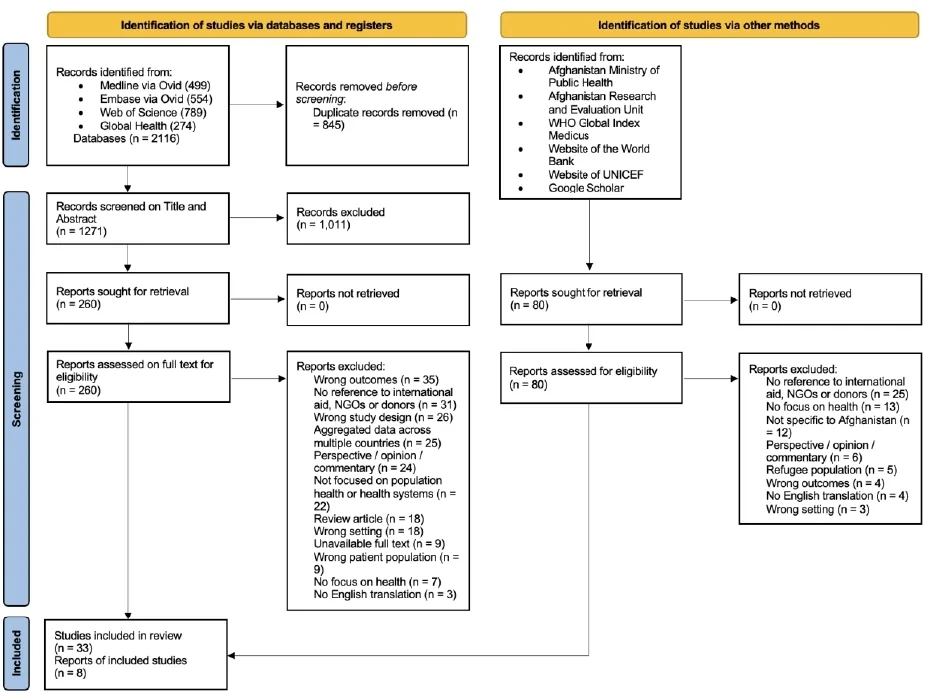
PRISMA 2020 flow diagram for study selection of databases and other sources. PRISMA, Preferred Reporting Items for Systematic Reviews and Meta-Analyses.

Stage 1 will involve screening titles and abstracts, followed by full-text screening at stage 2. Studies will be included if they are relevant to at least one research question. To minimise the risk of excluding relevant evidence, any records with reviewer disagreement at stage 1 will be retained for full-text screening.^26^ Disagreements at stage 2 will be resolved by a third independent reviewer.

### Critical appraisal

As this is a mixed-methods systematic review, study quality will be assessed using the Mixed Methods Appraisal Tool (MMAT), which is applicable to qualitative, quantitative and mixed-methods study designs.^27^ Each study will be independently appraised by two reviewers. Performance across MMAT domains will be reported for each study and used to describe study quality. Any disagreements will be resolved through discussion or by consultation with a third reviewer.

### Data extraction

Data will be extracted by both reviewers independently using a data extraction template in Microsoft Excel. Discrepancies will be resolved through discussion or by a third reviewer. Extracted data will include the authors, publication date, study type and aims, demographic characteristics of the study population, location (including hospital type, if applicable), sample size, domain of reduced international support (resources, staff, funding, etc), measurement tools and study outcomes.

### Outcomes

The primary outcome of this systematic review is the effect of reduced international aid on the healthcare system functioning and population health outcomes in Afghanistan. International aid will be measured using financial and non-financial indicators, including healthcare resources, equipment, staffing, infrastructure and direct service provision. Healthcare system functioning will be evaluated using indicators related to service availability and delivery, workforce capacity, resourcing, financing and governance, as well as population coverage. Population health outcomes will be assessed using measures such as disease prevalence, incidence, severity, mortality, morbidity and quality of life.

### Data synthesis

Given the expected heterogeneity in study design, populations, time periods and outcome measurement, meta-analysis is not feasible.^28^ As meta-analysis is not planned, statistical measures of heterogeneity will not be calculated. Heterogeneity will be explored narratively and through subgroup analyses. Statistical tests or funnel plots will not be used because no meta-analysis is planned. Hence, a narrative synthesis will be used to answer the research questions, given the aim of examining relationships across health-system domains and population groups.^29^ Data synthesis will follow the approach described by Popay *et al.*^28^

Background literature will be used to develop the initial conceptual model of how reductions in international aid affect the healthcare system’s ability to provide care according to the WHO health system building blocks framework.^30^ This framework comprises six components: service delivery; health workforce; health information systems; access to medicinal products, vaccines and technology; health system financing and leadership and governance. This model will be extended to consider how these system-level changes may influence health outcomes across different population groups.

Studies will then be grouped according to WHO building block categories to address the first research question. Key findings will be extracted within each domain, and patterns will be examined across studies to identify recurring themes, potential pathways, mediators, exceptions and contradictions. Study quality, assessed using the MMAT, will be considered when interpreting findings.^27^ Where studies do not explicitly attribute observed outcomes to reductions in international support, no causal link will be inferred.

To address the second research question, subgroup analyses will examine variation in health outcomes by population characteristics, including age, sex, location (including geographical constraints such as terrain and accessibility) and socioeconomic status, where reported.^31^ Relationships between system-level and population-level themes will then be examined to explore links between changes in system capacity and observed health outcomes.

The initial conceptual model will be refined in light of the synthesis findings and their certainty, according to the Grading of Recommendations, Assessment, Development and Evaluation (GRADE) framework.^32^ The final model will present an integrated account of how reductions in international aid have affected healthcare system functioning and which population groups appear most affected.

### Certainty of evidence

The certainty of evidence will be evaluated using the GRADE framework.^32^ Evidence will be categorised as ‘Very low’, ‘Low’, ‘Moderate’ or ‘High’ certainty. Assessments will be undertaken by two independent reviewers with disagreements resolved by a third reviewer. Publication bias will be considered as part of the GRADE assessment.

### Patient and public involvement

Patients and/or the public were not involved in the design, conduct, reporting or dissemination plan of this research.

### Reflexivity statement

RR has no personal or professional connection to Afghanistan. MSR is originally from Afghanistan and has several years’ experience in health research, while SAN is originally from Iran and has 7 years’ experience in global health research. The authors considered how their subjectivity, positionality and wider context could influence the interpretation of this systematic review’s findings. The review follows established systematic review methods to minimise bias.

## Discussion

### Review in light of existing literature

Evidence from other aid-dependent settings shows that reductions in external health support often affect multiple components of the health system simultaneously, especially financing, workforce capacity, service delivery and governance. Studies on sustainability of donor-supported health programmes showed that systems reliant on external support are vulnerable to service disruption when funding, technical support or parallel delivery structures are withdrawn.^33 34^ These effects are rarely uniform across the system and tend to reflect how deeply donor-funded activities are embedded within national planning, financing and delivery arrangements.

Studies of donor transition in HIV and immunisation programmes demonstrate these challenges. Evaluations of President’s Emergency Plan for AIDS Relief (PEPFAR) transition in South Africa and other settings report risks to staffing, continuity of services and programme monitoring where donor-supported posts and implementing partners were not absorbed into domestic systems.^35^ Similarly, analyses of countries transitioning away from Global Alliance for Vaccines and Immunisation support highlight financing gaps, procurement challenges and governance pressures when domestic replacement funding is delayed or incomplete.^36^ These reports suggest that reductions in international aid can translate into downstream health effects through indirect pathways operating across multiple system domains.

### Implications for research and practice

This review addresses a gap in the literature on the impact of reduced international aid on both the healthcare system’s ability to provide care and population health outcomes in Afghanistan. It may inform policymakers, international donors, non-governmental organisations and the Afghanistan Ministry of Public Health about the effects of reduced international aid on healthcare system functioning and population health. It may also guide future resource allocation and efforts to strengthen health system sustainability.

### Strengths and limitations

A strength of this review is its focus on how reductions in international aid affect specific components of the healthcare system and associated population health outcomes. Structuring the synthesis using the WHO health system building blocks allows system-level changes to be examined in a consistent and transparent manner and linked to reported health effects across population groups. The inclusion of qualitative, quantitative and mixed-methods studies, together with grey literature, allows the review to capture both outcome data and operational evidence from a fragile and conflict-affected setting where peer-reviewed evidence alone is likely to be incomplete.

The review is likely to be limited by heterogeneity in study design, outcome measurement and data availability, particularly following August 2021, when access to routine data collection has been disrupted. Some health system domains and population groups may therefore be under-represented. As inclusion was restricted to studies with full texts available in English, we may have excluded relevant evidence published in Dari, Pashto or other languages. Although study quality and certainty will be assessed systematically, conclusions will depend on the scope and depth of available evidence. Findings may have limited direct applicability beyond Afghanistan, given its political and humanitarian context. However, the review may identify system-level patterns relevant to other aid-dependent health systems. As a narrative synthesis, the analysis cannot support statistical causal inference and should be interpreted accordingly.^28^

A structured risk assessment and contingency plan was developed to identify potential sources of bias, heterogeneity and data limitations across study selection, extraction and synthesis. These risks and planned mitigation strategies are presented in online supplemental file 2.

### Review status

At protocol resubmission, the literature searches, study selection and data extraction had been completed, and analysis was ongoing.

### Ethics and dissemination

This study does not require ethical approval as it involves secondary data analysis. Findings will be disseminated through peer-reviewed publications to inform international policy.

## Supplementary material

10.1136/bmjopen-2026-117725 online supplemental file 1

10.1136/bmjopen-2026-117725 online supplemental file 2
