# Supplementary material for: International aid dependence and healthcare sustainability in Afghanistan: study protocol for a systematic review
Source: BMJ Open. 2026 Nov 4;16(11):e117725. doi: 10.1136/bmjopen-2026-117725 (PMC13735327; doi:10.1136/bmjopen-2026-117725)
Supplement: Supplementary file 1 [file bmjopen-16-11-s002.pdf]

Supplemental file 2: Risk assessment and mitigation strategies

Table 4 Risk assessment, split by category, and contingency plan

**Category**	**Risk Assessment**	**Contingency Plan**
Study Selection	Publication bias as studies showing mild or insignificant results are less likely to be published.	Assess risk of publication bias using GRADE framework. Search grey literature to identify unpublished relevant literature.
	Missing relevant literature due to inadequate search.	Allocate a significant portion of the timeline on literature searching, consult with expert librarian to refine search.
Data Extraction	Heterogeneity within aims, methodologies, sample populations, and results.	Conduct a narrative synthesis and use subgroup analysis to infer differences between system elements or demographics and relationships between subgroups.
	Biases from study design, including recall bias, social desirability bias, measurement bias, attrition bias, and confirmation bias.	Critically appraise studies using Mixed Methods Appraisal Tool (MMAT)
	Low quality studies.	Critically appraise overall evidence using GRADE framework, consider scores when amending final conceptual model.
	Demographics or health system components missing data due to lack of studies.	Infer potential links between them and other subgroups, acknowledge this as a limitation in the Discussion section.
Data Synthesis	Loss of true meaning of qualitative study's results due to multiple rounds of interpretation.	Develop a thorough data synthesis plan that is grounded in the original data, validate methods with supervisor.
	Lack of generalisability due to Afghanistan's unique situation.	Draw on potential commonalities with similar countries and develop translatable, pragmatic conclusions.
