# Supplementary material for: International aid dependence and healthcare sustainability in Afghanistan: study protocol for a systematic review
Source: BMJ Open. 2026 Nov 4;16(11):e117725. doi: 10.1136/bmjopen-2026-117725 (PMC13735327; doi:10.1136/bmjopen-2026-117725)
Supplement: Supplementary file 2 [file bmjopen-2026-117725.reviewer_comments.pdf]

PEER REVIEW HISTORY

BMJ Open publishes all reviews undertaken for accepted manuscripts. Reviewers are asked to complete a checklist review form and are provided with free text boxes to elaborate on their assessment. These free text comments are reproduced below.

ARTICLE DETAILS

Title (Provisional)

International aid dependence and healthcare sustainability in Afghanistan: study protocol for a systematic review

Authors

Rahman, Ramim; Nejadghaderi, Seyed Aria; Razai, Mohammad Sharif

---

VERSION 1 - REVIEW

**Reviewer**	**1**
**Name**	**Zwi, Karen**
**Affiliation**	**University of New South Wales**
**Date**	**24-Mar-2026**
**COI**	**None**

Excellent protocol addressing the important issue of international aid dependence and healthcare sustainability. No significant issue to address.

Minor issues:

-include total population of Afghanistan

- Searches P9L15 (page, line)

- conceptual model P11L31

- address tense P11L50

- write GAVI in full P12L40

**Reviewer**	**2**
**Name**	**Norii, Tatsuya**
**Affiliation**	**University of New Mexico - Albuquerque, Emergency Medicine**
**Date**	**12-Jul-2026**

COI **None**

Summary:

This protocol outlines a planned systematic review aimed at synthesizing evidence on the effects of reduced international aid on Afghanistan's healthcare system functioning and population health outcomes. Overall, the manuscript is very well written, and the proposed methods are technically sound. Nevertheless, I have several questions and comments regarding the protocol that may help further strengthen the study and enhance the clarity of its methodology.

Specific comments:

1. The current status of the project is somewhat unclear. The manuscript indicates that the database search was completed in January 2026, while the gray literature search has not yet been initiated. Please clarify the rationale for conducting these components of the search strategy at different time points and discuss whether the delay could affect the comprehensiveness or currency of the review.

2. In the Methods section, I could not find a description of any language restrictions applied to the search strategy. Do the authors plan to limit the search to studies published in English only? If so, have the authors considered including studies published in other languages when an English abstract is available? Language restrictions may introduce language bias and potentially exclude relevant evidence, particularly given the international nature of the topic. Therefore, I encourage the authors to clarify their planned approach to non-English publications and to provide a rationale for any language restrictions that will be applied.

Minor comments:

Page 3, line 37: Consider spelling out WHO (World Health Organization) at its first mention.

Page 8, line 1: "SPIDER" was already spelled out on the previous page (Page 7). Consider using the abbreviation alone here to avoid unnecessary repetition.

VERSION 1 - AUTHOR RESPONSE

Reviewer: 1

Dr. Karen Zwi, University of New South Wales, Sydney Children\'s Hospitals Network Randwick and Westmead

Comments to the Author:

Excellent protocol addressing the important issue of international aid dependence and healthcare sustainability. No significant issue to address.

Minor issues:

-include total population of Afghanistan

- Searches P9L15 (page, line)

- conceptual model P11L31

- address tense P11L50

- write GAVI in full P12L40

Response: Thank you very much for your feedback and kind words. We have addressed all of the issues in our revised manuscript.

Reviewer: 2

Dr. Tatsuya Norii, University of New Mexico - Albuquerque

Comments to the Author:

Summary:

This protocol outlines a planned systematic review aimed at synthesizing evidence on the effects of reduced international aid on Afghanistan's healthcare system functioning and population health outcomes. Overall, the manuscript is very well written, and the proposed methods are technically sound. Nevertheless, I have several questions and comments regarding the protocol that may help further strengthen the study and enhance the clarity of its methodology.

Specific comments:

1. The current status of the project is somewhat unclear. The manuscript indicates that the database search was completed in January 2026, while the gray literature search has not yet been initiated. Please clarify the rationale for conducting these components of the search strategy at different time points and discuss whether the delay could affect the comprehensiveness or currency of the review.

Response: Thank you. We have clarified that the database searches were conducted in January 2026 and the grey literature search in June 2026. The searches were undertaken sequentially. Screening of the database records informed the subsequent searches of organisational websites and Google Scholar by clarifying the types of reports most relevant

to the review. Conducting the grey literature search later allowed us to identify more recent relevant organisational reports. We have added a review status at the end of the manuscript.

2. In the Methods section, I could not find a description of any language restrictions applied to the search strategy. Do the authors plan to limit the search to studies published in English only? If so, have the authors considered including studies published in other languages when an English abstract is available? Language restrictions may introduce language bias and potentially exclude relevant evidence, particularly given the international nature of the topic. Therefore, I encourage the authors to clarify their planned approach to non-English publications and to provide a rationale for any language restrictions that will be applied.

Response: Thank you. We have clarified the English-language eligibility criterion and its rationale in the *Methods*. Full texts are restricted to English to make sure consistent independent screening, data extraction and appraisal across reviewers. We have acknowledged the potential for language bias as a limitation in the *Discussion*.

Minor comments:

Page 3, line 37: Consider spelling out WHO (World Health Organization) at its first mention.

Response: Thank you. This is done as suggested.

Page 8, line 1: "SPIDER" was already spelled out on the previous page (Page 7). Consider using the abbreviation alone here to avoid unnecessary repetition.

Response: Thank you. This is corrected as suggested.
