# Supplementary material for: International aid dependence and healthcare sustainability in Afghanistan: study protocol for a systematic review
Source: BMJ Open. 2026 Nov 4;16(11):e117725. doi: 10.1136/bmjopen-2026-117725 (PMC13735327; doi:10.1136/bmjopen-2026-117725)
Supplement: Supplementary file 3 [file bmjopen-16-11-s001.docx]

# Supplemental file 1: Search strategies for each database

| **Table 1** Number of papers per database and in total | |
| --- | --- |
| **Database** | **Number of Hits** |
| Medline via Ovid | 499 |
| Embase via Ovid | 554 |
| Global Health | 274 |
| Web of Science | 789 |
| Total with duplicates | 2116 |
| Total without duplicates | 1610 |

## Medline and Embase

| Table 2 Keyword and subject search for Medline and Embase | | | |
| --- | --- | --- | --- |
|  | **Keyword** | **Medline** | **Embase** |
| 1 | exp Afghanistan/ | 4118 | 8252 |
| 2 | Afghan*.mp. | 11269 | 13031 |
| 3 | 1 or 2 | 11269 | 13031 |
| 4 | exp Health Services Administration/ | 4088843 | 8393197 |
| 5 | Capacity Building/ | 4055 | 9898 |
| 6 | Health Communication/ | 3806 | 99990 |
| 7 | exp Health Facilities/ | 959866 | 2371489 |
| 8 | Health Workforce/ | 15207 | 16090 |
| 9 | exp Health Personnel/ | 673689 | 2349326 |
| 10 | exp Health Services/ | 2615905 | 8393197 |
| 11 | Public Health Infrastructure/ | 23 | 1642 |
| 12 | exp Health Planning/ | 391512 | 123946 |
| 13 | Health Care Coalitions/ | 2375 | 145016 |
| 14 | exp Health Policy/ | 139419 | 247550 |
| 15 | 4 or 5 or 6 or 7 or 8 or 9 or 10 or 11 or 12 or 13 or 14 | 6270705 | 10493757 |
| 16 | (health system* or healthcare system* or health* inequal* or health* workforce or staffing or health* personnel or health* facilities or hospital* or clinic* or primary care or medical equipment or hospital resourc* or medical resourc* or health manag* or health govern* or health leader* or delivery of health* or patient management or health policy).mp. | 9016903 | 18782729 |
| 17 | 15 or 16 | 11944524 | 21544669 |
| 18 | exp Mortality/ | 449606 | 1641019 |
| 19 | exp Morbidity/ | 718105 | 503314 |
| 20 | exp Infant Mortality/ | 32946 | 23654 |
| 21 | exp Life Expectancy/ | 46158 | 72883 |
| 22 | exp Health Status/ | 534128 | 409862 |
| 23 | Child Health/ | 6171 | 41204 |
| 24 | Infant Health/ | 1532 | 41204 |
| 25 | Reproductive Health/ | 6479 | 31244 |
| 26 | exp Women’s Health/ | 33711 | 38092 |
| 27 | Incidence/ | 330571 | 743296 |
| 28 | Prevalence/ | 384644 | 1201127 |
| 29 | exp “Quality of Health Care”/ | 8981008 | 5499272 |
| 30 | exp Communicable Diseases/ | 603309 | 1230349 |
| 31 | exp Population Health/ | 41575 | 8779 |
| 32 | exp Health Inequities/ | 49621 | 56871 |
| 33 | 18 or 19 or 20 or 21 or 22 or 23 or 24 or 25 or 26 or 27 or 28 or 29 or 30 or 31 or 32 | 9791751 | 9272715 |
| 34 | (immuni* or vaccin* or maternal health* or women* health* or child* health* or adolescent* health* or infant* health* or reproductive health* or maternal mortality or child* mortality or infant mortality or neonatal mortality or perinatal mortality or mortality or morbidity or quality of care or health outcome* or disease burden or incidence or prevalence or survival or life expectancy or communicable disease* or infectious disease* or disease manag* or health inequalit* or health inequit* or health* access*).mp. | 6052340 | 9068119 |
| 35 | 33 or 34 | 12796054 | 13384192 |
| 36 | 17 or 35 | 17589351 | 25147900 |
| 37 | (humanitarian or humanitarian assistance or humanitarian aid or foreign aid or international aid or foreign assistance or international support or external support or donor* or funding or budget* or expenditure* or development assistance or aid cut* or fund* cut* or funding suspen* or withdraw* or sanction* or financial restriction* or health system strain or healthcare system strain or health system collapse or healthcare system collapse or service disruption or system disruption or fragil* or crisis).mp. | 1011133 | 1812121 |
| 38 | 3 and 36 and 37 | 697 | 1022 |
| 39 | Conference proceeding.pt. | 0 | 0 |
| 40 | Conference abstract.pt. | 0 | 5858433 |
| 41 | Conference.pt. | 0 | 6648809 |
| 42 | Conference.mp. | 104615 | 3843264 |
| 43 | Editorial.pt. | 746652 | 859042 |
| 44 | Note.pt. | 0 | 1033434 |
| 45 | Letter.pt. | 1324318 | 1395547 |
| 46 | Review.pt. | 3445245 | 3477710 |
| 47 | Review.ti,ab. | 2629623 | 3227889 |
| 48 | Meta-analysis.pt. | 224165 | 0 |
| 49 | Meta-analysis.ti,ab. | 320542 | 405205 |
| 50 | Meta analysis.pt. | 224165 | 0 |
| 51 | Meta analysis.ti,ab. | 320542 | 405205 |
| 52 | 39 or 40 or 41 or 42 or 43 or 44 or 45 or 46 or 47 or 48 or 49 or 50 or 51 | 6760218 | 14555666 |
| 53 | 38 not 52 | 530 | 573 |
| 54 | limit 53 to yr=”2000 -Current” | 499 | 554 |

## Web of Science and Global Health

| **Table 3** Keyword search for Web of Science and Global Health according to our SPIDER framework | |
| --- | --- |
| **SPIDER category** | **Keywords** |
| Sample | Afghan* |
| AND | |
| Phenomenon of Interest | (health system* OR healthcare system* OR health* servic* OR health* workforce OR staffing OR health* personnel OR health* facilities OR hospital* OR clinic* OR primary care OR medical equipment OR hospital resourc* OR medical resourc* OR health manag* OR health govern* OR health leader* OR delivery of health* OR patient management OR health policy OR immuni* OR vaccin* OR maternal health* OR women* health* OR child* health* OR adolescent* health* OR infant* health* OR reproductive health* OR maternal mortality OR child* orality OR infant mortality OR neonatal mortality OR perinatal mortality OR mortality OR morbidity OR quality of care OR health outcome* OR disease burden OR incidence OR prevalence OR survival OR life expectancy OR communicable disease* OR infectious disease* OR disease manag* OR health inequalit* OR health inequit* OR health* access*) |
| NOT | |
| Design | (review OR conference OR editorial OR letter OR note OR meta-analysis OR meta analysis) |
| AND | |
| Evaluation | (humanitarian OR humanitarian assistance OR humanitarian aid OR foreign aid OR international aid OR foreign assistance OR international support OR external support OR donor* OR funding OR budget* OR expenditure* OR development assistance OR aid cut* OR fund* cut* OR funding suspen* OR withdraw* OR sanction* OR financial restriction* OR health system strain OR healthcare system strain OR health system collapse OR healthcare system collapse OR service disruption OR system disruption OR fragil* OR crisis) |

Additionally, a filter of publication year from 2000 onwards was applied.

Given the inclusion of qualitative, quantitative and mixed-methods studies, we decided to not include the ‘Research type’ (R) component of our SPIDER framework into our search strategy, as this would unnecessarily restrict our search and may remove relevant literature. ‘Design’ (D) was included to limit our search to primary research only. Initial scoping searches were conducted in all databases using the ‘Sample’ (S) and ‘Phenomenon of Interest’ (PI) components. This produced a very large number of results that would be unfeasible to screen considering our timeline and resources. Many of these papers were also not relevant to our study as they did not discuss international aid reductions as a potential driver for their observed results. Hence, Evaluation (E) was included in our search strategies for all databases.
